# Massive obstetric hemorrhage during cesarean section in a patient after conception by frozen-thawed embryo transfer: a case report

**DOI:** 10.1186/s40981-019-0308-0

**Published:** 2020-01-06

**Authors:** Mai Ito, Kensuke Oshita, Kazuyuki Tanaka, Masato Hara, Teruyuki Hiraki

**Affiliations:** Department of Anesthesiology, Kurume University School of Medicine, 67 Asahi-machi, Kurume, Fukuoka, 830-0011 Japan

**Keywords:** Cesarean section, Frozen-thawed embryo transfer, Placenta accreta, Systemic lupus erythematosus

## Abstract

**Background:**

Placenta accreta is a major cause of massive obstetric hemorrhage during cesarean section. In recent years, pregnancy by in vitro fertilization-embryo transfer has been reported as a risk factor for placenta accreta.

**Case presentation:**

A 36-year-old G1P0 woman with systemic lupus erythematosus became pregnant by frozen-thawed embryo transfer. Emergency cesarean section was performed under general anesthesia due to the diagnosis of non-reassuring fetal status. The placenta invaded the myometrium and completely covered the entire anterior uterine wall. Following birth, 3000 mL of blood loss required rapid fluid infusion and blood transfusion. Total hysterectomy was performed because the placenta could not be separated from the uterine wall. Histological examination revealed placenta accreta/increta.

**Conclusions:**

When performing cesarean section on patients who have undergone frozen-thawed embryo transfer, preoperative examinations to assess for placenta accreta should be performed, and the anesthetic management should include sufficient planning for massive obstetric hemorrhage.

## Background

Placenta accreta is a major obstetric complication in which the chorionic villi invade the myometrium, causing part of or the entire placenta to become strongly attached to the uterine wall. This makes placental detachment difficult, leading to massive hemorrhage following delivery or perinatal emergency hysterectomy. Risk factors include placenta previa, prior cesarean section, and prior uterine surgery. Assisted reproduction technology (ART) treatments, especially in vitro fertilization (IVF), have been recently reported as a new risk factor for placenta accreta [1, 2].

We report a primigravida with systemic lupus erythematosus (SLE) who became pregnant by frozen-thawed embryo transfer (FET) and underwent hysterectomy due to poor control of unexpected massive hemorrhage caused by placenta accreta during cesarean section. There have only been a few reports describing the association between placenta accreta and SLE [3, 4].

## Case presentation

The patient was a 36-year-old woman (152 cm, 52 kg) who was G1P0 and had no history of gynecological surgery. She was diagnosed with SLE at the age of 19 and was maintained on prednisolone 15 mg daily. She also received subcutaneous injections of 2000 units of low-molecular-weight heparin for suspected antiphospholipid antibody syndrome. She had undergone infertility treatment for 8 years and had five ART treatments at another medical institution. The patient successfully became pregnant by FET at our institution. Although the pregnancy progressed smoothly, at 36 weeks and 3 days of gestation, emergency cesarean delivery was performed due to the diagnosis of non-reassuring fetal status. Preoperative ultrasound examination revealed a massive placenta covering the lower half of the uterine body; however, these findings were not considered placenta previa. Parts of the myometrium had become thin and placental lacunae were noted. Furthermore, the boundary between the myometrium and placenta was indistinct.

**Fig. 1:**
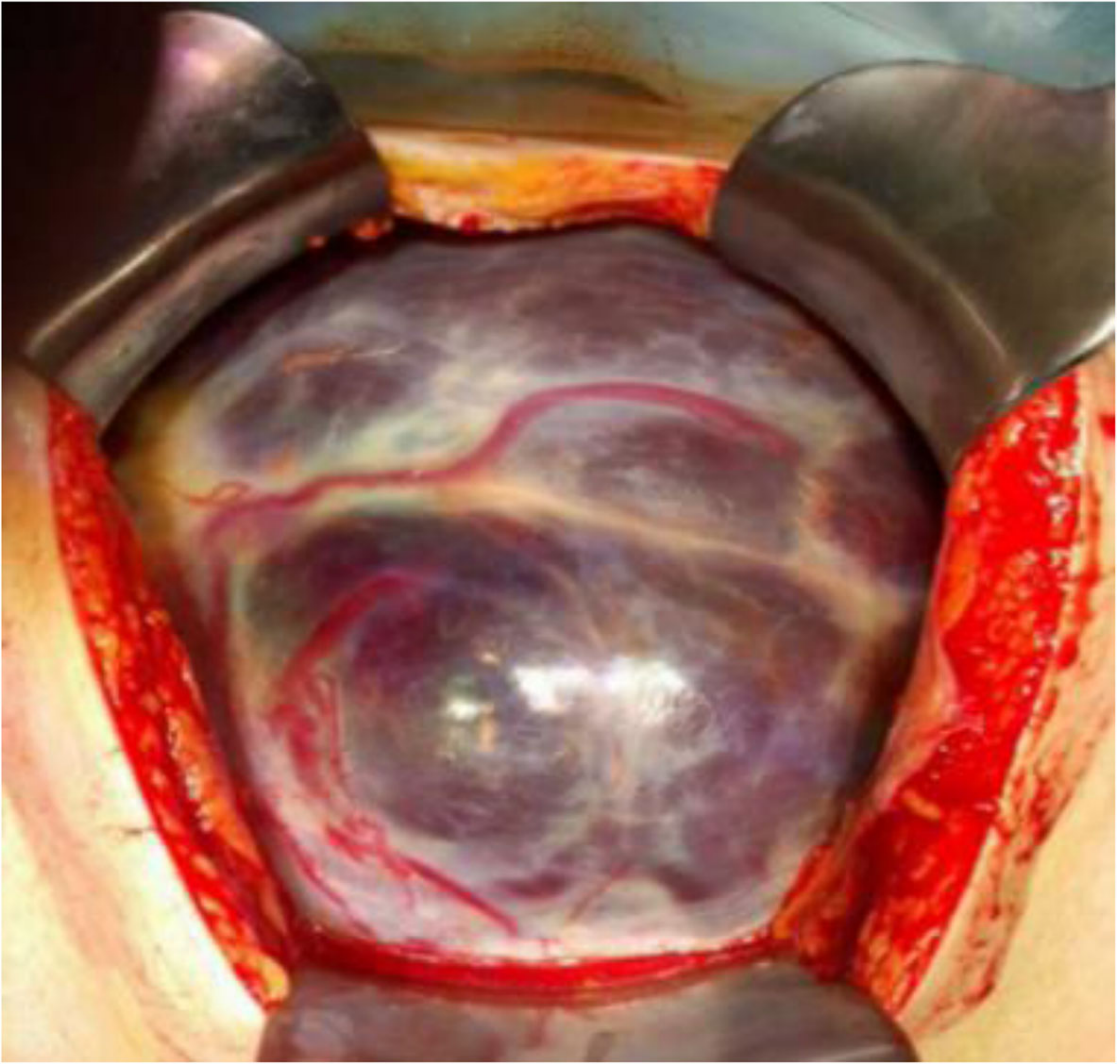
Uterus with placenta accreta visible at the time of laparotomy. The placenta was bulging through a thin myometrium on the anterior uterine wall. A massive placenta covers the anterior uterine wall with increased vascularity. The fetus was delivered through the placenta

General anesthesia was employed because she had already received subcutaneous injection of low-molecular-weight heparin on the day of the operation. Anesthesia was induced by propofol 120 mg, remifentanil 0.3 μg/kg/min, and rocuronium 50 mg and maintained with target controlled infusion of propofol at 2–3 μg/ml and remifentanil 0.1–0.25 μg/kg/min with 60% oxygen after tracheal intubation. Intraoperatively, the myometrium of the anterior uterine wall was thin and the placenta, which extended over the entire anterior uterine wall, was observed through it (Fig. 1). Because massive hemorrhage was expected, additional intravenous access (18-gauge) was obtained, a radial arterial catheter was placed, and blood products were prepared. The fetus was delivered through the placenta. Following birth, systolic blood pressure rapidly dropped to approximately 60 mmHg, and 3000 mL of blood loss required rapid fluid infusion and blood transfusion. Emergency total hysterectomy was performed because the placenta was firmly attached to the uterine wall and was unable to separate, causing massive hemorrhage. Intraoperative blood loss was 5860 mL, including the amniotic fluid. She was transfused 10 units of packed red blood cells, 6 units of fresh frozen plasma, and 10 units of platelet concentrate. Apgar scores of the newborn were 6 and 8 at 1 and 5 min, respectively. The patient and the baby were both discharged without complications. Histological examination demonstrated multiple areas where the myometrium had become markedly thin and the placental villi had directly invaded the myometrium without the decidual layer, consistent with placenta increta.

## Discussion

Placenta accreta is defined as a placenta that in whole or in part invades the uterine wall and is inseparable from it [5]. Placenta accreta is one of the most serious obstetric complications and is associated with an increased risk of massive hemorrhage during cesarean section. Peripartum emergency hysterectomy is often required to control massive hemorrhage. Risk factors include a history of a cesarean section, placenta previa, maternal age, and a history of curettage [6].

In recent years, ART treatments, especially IVF, have been reported as a new risk factor for placenta accreta [1, 2]. Esh-Broder et al. reported that the rate of placenta accreta in the IVF group was 13.2-fold higher than that in the spontaneous pregnancy group [1]. FET in particular leads to a higher incidence of placenta accreta than fresh embryo transfer (0.27% and 0.09%, respectively) [7–9]. As our patient did not have conventional risk factors for placenta accreta, pregnancy achieved by FET may have been one of the risk factors for placenta accreta in this case. Anesthetic management for massive obstetric hemorrhage should be prepared for patients who underwent FET, even without a history of cesarean section or other uterine surgeries, because placenta accreta may only be diagnosed at the time of delivery. In our case, a preoperative definitive diagnosis of placenta accreta was unable to be made, although it was suggested by preoperative ultrasound imaging findings. Magnetic resonance imaging may have helped to confirm the diagnosis in this case. Also, measures to decrease intraoperative blood loss, e.g., intra-aortic balloon occlusion or iliac artery balloon catheter placement, should have been considered preoperatively.

This patient also had a background of SLE. In a study of 25 pregnant women with SLE complications, underdevelopment and dysfunction of the placental villi were observed and were likely caused by reduced blood flow in the intervillous space through the myometrium due to vascular abnormalities of the placenta [10]. Similar to the present study, hysterectomy was performed during cesarean section because of placenta accreta and myometrial thinning in a pregnancy with SLE complications [3, 4]. The relationships among SLE, myometrial thinning, and placenta accreta have not been elucidated. A larger number of cases and more data are necessary to determine such a relationship.

In conclusion, we report the case of a primigravida with a background of SLE who became pregnant by FET. Massive hemorrhage developed following delivery and emergency total hysterectomy was performed due to placenta accreta. When performing cesarean section on patients who have undergone FET, preoperative examinations to assess for placenta accreta should be performed, and the anesthetic management should include sufficient planning for massive hemorrhage. Further cases and data are necessary to clarify the association between placenta accreta and SLE.

## Data Availability

The data in this case report are available from the corresponding author upon reasonable request.

## Abbreviations

ART: Assisted reproductive technology
FET: Frozen-thawed embryo transfer
IVF: In vitro fertilization
SLE: Systemic lupus erythematosus
